# Protein-based SARS-CoV-2 spike vaccine booster increases cross-neutralization against SARS-CoV-2 variants of concern in non-human primates

**DOI:** 10.1038/s41467-022-29219-2

**Published:** 2022-03-31

**Authors:** Vincent Pavot, Catherine Berry, Michael Kishko, Natalie G. Anosova, Dean Huang, Tim Tibbitts, Alice Raillard, Sylviane Gautheron, Cindy Gutzeit, Marguerite Koutsoukos, Roman M. Chicz, Valerie Lecouturier

**Affiliations:** 1 Sanofi, Marcy l’Etoile, France; 2 Sanofi, Cambridge, MA USA; 3 GSK, Rixensart, Belgium; 4 GSK, Wavre, Belgium

**Keywords:** Protein vaccines, Viral infection, SARS-CoV-2, Preclinical research

## Abstract

The emergence of severe acute respiratory syndrome coronavirus 2 (SARS-CoV-2) variants that partly evade neutralizing antibodies raises concerns of reduced vaccine effectiveness and increased infection. We previously demonstrated that the SARS-CoV-2 spike protein vaccine adjuvanted with AS03 (CoV2 preS dTM-AS03) elicits robust neutralizing antibody responses in naïve subjects. Here we show that, in macaques primed with mRNA or protein-based subunit vaccine candidates, one booster dose of CoV2 preS dTM-AS03 (monovalent D614 or B.1.351, or bivalent D614 + B.1.351 formulations), significantly boosts the pre-existing neutralizing antibodies against the parental strain from 177- to 370-fold. Importantly, the booster dose elicits high and persistent cross-neutralizing antibodies covering five former or current SARS-CoV-2 variants of concern (Alpha, Beta, Gamma, Delta and Omicron) and, unexpectedly, SARS-CoV-1. Interestingly, we show that the booster specifically increases the functional antibody responses as compared to the receptor binding domain (RBD)-specific responses. Our findings show that these vaccine candidates, when used as a booster, have the potential to offer cross-protection against a broad spectrum of variants. This has important implications for vaccine control of SARS-CoV-2 variants of concern and informs on the benefit of a booster with the vaccine candidates currently under evaluation in clinical trials.

## Abstract (web summary)

Emerging SARS-CoV-2 variants of concern (VOCs) raise questions on vaccine effectiveness. Here, the authors show that an adjuvanted protein-based SARS-CoV-2 spike vaccine booster increases cross-neutralization of VOC, including omicron, in non-human primates.

## Introduction

Emergence of severe acute respiratory-syndrome coronavirus-2 (SARS-CoV-2) variants of concern (VOC), such as Alpha (B.1.1.7), Beta (B.1.351), and Delta (B.1.617.2) in late 2020 and lastly Omicron (B.1.1.529) in November 2021 have sparked concerns due to their higher transmissibility and/or pathogenicity^1–4^. These VOC harbor specific mutations in the spike proteins impacting the transmission and antigenicity reflecting growing immune pressure at positions shown to involve antibody recognition^5^. Some VOC (Beta, Gamma, Delta, and Omicron) have also demonstrated partial evasion of natural and vaccine-elicited neutralizing antibodies^6–10^ and are associated with reduction of vaccine effectiveness^11–13^. In response to the rapid evolution of SARS-CoV-2 and the global circulation of VOC since the end of 2020, vaccines are being developed using a modified spike antigen containing the mutations identified in Beta, Delta, and Omicron variants (NCT04785144)^14–16^. This strategy is based on the influenza-vaccine model where the vaccine is modified yearly according to the epidemiology, likewise, a combination of spike antigens covering multiple cocirculating strains may be necessary. In early 2021, the Beta spike (B.1.351) was selected, as it displayed the greatest breakthrough infections against the parental (D614) vaccines^11,12^, before Omicron was shown to cause even higher levels of vaccine escape. Considering the risk of waning immunity after natural infection or immunization and the risk of vaccine escape by emerging variants, the key attributes of future vaccines will be the ability to boost, prolong, and broaden protective immunity against new emerging variants.

Here we formulated soluble prefusion-stabilized spike trimers (CoV2 preS dTM) with the well-characterized adjuvant AS03, an oil-in-water emulsion composed of α-tocopherol, squalene, and polysorbate 80^17^. AS03 has been shown to potently induce antibody responses, increase vaccine durability, promote heterologous strain cross-reactivity, and has dose-sparing effects^18,19^. We previously reported that CoV2 preS dTM-AS03 (D614) confers protective efficacy against SARS-CoV-2 (D614 strain) challenge in nonhuman primates (NHPs) and the vaccine-induced IgG mediated protection from SARS-CoV-2 challenge following passive transfer to hamsters^20^. We also showed safety and immunogenicity of an optimized vaccine formulation in humans in a phase-2 clinical trial^21^ and the vaccine formulation is being assessed as a booster vaccine (NCT04762680) and for efficacy (NCT04904549) in phase-3 clinical studies.

In this work, the objective was to assess a protein-based subunit vaccine booster in macaques vaccinated 7 months before with either mRNA–LNP or subunit CoV2 preS dTM-AS03 Sanofi vaccine candidates. Various formulations, namely AS03-adjuvanted parental (D614), variant (B.1.351), and bivalent (D614 + B.1.351) CoV2 preS dTM or non-adjuvanted CoV2 preS dTM (B.1.351), were evaluated for their ability to boost neutralizing antibodies (NAbs) against the parental strain and to induce cross-neutralization against the five VOC described so far (Alpha, Beta, Gamma, Delta, and Omicron) and SARS-CoV-1 (from the 2003 outbreak) to further explore the breadth of neutralization.

## Results

### Boosting with homologous or heterologous formulations of CoV2 preS dTM led to significant increase in neutralizing antibodies against parental SARS-CoV-2, with a significant AS03-adjuvant effect

**Fig. 1:**
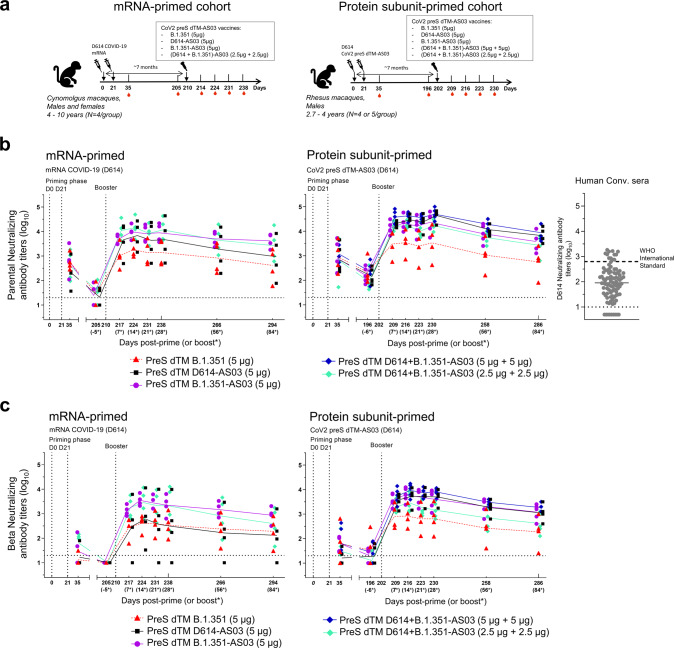
Kinetics of booster-neutralizing antibody responses (parental and Beta) in mRNA- or subunit-primed macaques. **a** Schematic representation of the study schedule. In mRNA-primed cohort, 4 groups of 4 cynomolgus macaques were immunized intramuscularly with mRNA COVID-19 (D614) vaccine candidates on day 0 (D0) and on day 21 (D21). In subunit-primed cohort, 5 groups of 4–5 rhesus macaques were immunized intramuscularly with CoV2 preS dTM-AS03 (D614) vaccine candidates on D0 and D21. Both cohorts were boosted 7 months post-dose 1 with nonadjuvanted monovalent (B.1.351) CoV2 preS dTM (*n* = 4) or AS03-adjuvanted monovalent (D614 or B.1.351) (*n* = 5) or bivalent (D614 + B.1.351) CoV2 preS dTM (*n* = 5). Humoral immune responses were assessed at different timepoints throughout the study. **b** Pseudovirus-neutralizing antibody titers against the parental SARS-CoV-2 and **c** Beta variant were assessed at different timepoints after the priming phase and after the booster immunization in macaques. Individual macaque data are shown. Connecting lines indicate mean responses and horizontal dotted lines the limits of quantification of the assay. *timepoints relative to boosters. A human convalescent panel of sera (Human Conv. sera) (*n* = 93) was assayed against D614 and is plotted as a comparator. Dotted line represents the World Health Organization (WHO) International Standard for anti-SARS-CoV-2 immunoglobulin (human) NIBSC code: 20/136.

Following primary vaccination with either mRNA–LNP or CoV2 preS dTM-AS03 (Fig. 1a), we assessed the neutralizing antibody (NAb) responses against the parental D614G strain. Using a GFP-based pseudovirus-neutralization assay, the mean NAb titers at day 35 (D35, 2 weeks post-dose 2) against the parental pseudovirus were 2.6 log_10_ for the mRNA-primed cohort and 2.9 log_10_ for the subunit-primed cohort (Fig. 1b). Seven months post prime (baseline pre-boost), mean parental NAb titers declined to 1.4 log_10_ for the mRNA-primed cohort and to 2.2 log_10_ for the subunit-primed cohort. For comparison, the mean D614 NAb titer in a panel of 93 human convalescent sera (collected within 3 months after positive PCR test) was 2.0 log_10_ (D614 and D614G NAb titers were shown to be similar in a concordance analysis, Supplementary Fig. 1) and the WHO International Standard for anti-SARS-CoV-2 immunoglobulin (human) (National Institute for Biological Standards and Control (NIBSC) code: 20/136) had a titer of 2.8 log_10_.

A booster dose with non-adjuvanted CoV2 preS dTM (B.1.351) significantly increased parental NAb titers compared with baseline as soon as 7 days after injection, reaching a plateau between 14 and 28 days in both mRNA-primed (mean of 3.3 log_10_) and subunit-primed (mean of 3.5 log_10_) cohorts (*p*-value < 0.001), and then slightly decreased up to 3 months post booster (mean of 2.6 and 2.8 log_10_ in mRNA-primed and subunit-primed cohorts, respectively). As in each cohort, macaques were randomized for their baseline titers 6 months post-primary vaccination and peak titers post- prime (D35), we analyzed the fold-increase of NAb titers 14 days post-booster compared with baseline or post-prime. Compared with baseline, mean increase in NAbs were 60-fold (*p*-value < 0.001) in mRNA-primed and 16-fold (*p*-value < 0.001) in subunit-primed cohorts. Of note, baseline titers in the mRNA cohort were lower than in the subunit cohort. Compared with post-prime, the mean parental NAb titers 14 days post-booster were also significantly higher than the peak titer at D35 (3.2-fold, *p*-value < 0.05 and 3.8-fold, *p*-value < 0.01 for mRNA-primed and subunit-primed cohorts, respectively).

A booster dose with CoV2 preS dTM-AS03 adjuvanted vaccine candidates (monovalent or bivalent formulations) also significantly increased parental NAb titers compared with baseline in both cohorts as early as 7 days after injection, and by 14 days, the mean titers were 4.0 log_10_ in mRNA-primed (mean fold increase of 370, *p*-value < 0.001) and 4.4 log_10_ in subunit-primed (mean fold-increase of 177, *p*-value < 0.001) animals. The titers were stable up to D28, and then slightly declined up to D84 (mean titers at 3.3 log_10_ and 3.7 log_10_ in the mRNA- and subunit-primed cohorts, respectively).

In both the mRNA- and subunit-primed cohorts, after the AS03-adjuvanted booster, the parental NAb titers (D7–D28 post-boost) were significantly higher than the peak titers post-priming vaccination at D35 (mean fold increase of 21 in the mRNA-primed cohort and 31 in the subunit-primed cohort, *p*-value < 0.001). There were no statistically significant differences in parental NAb titer increase (from D35 post primary vaccination or from baseline pre-booster) between the various AS03-adjuvanted vaccine formulations.

In both cohorts, a significant adjuvant effect was observed on the fold increase from baseline to 2 weeks post-booster with CoV2 preS dTM-AS03 (B.1.351) vaccine compared with nonadjuvanted CoV2 preS dTM (B.1.351). AS03 induced a 6.5-fold and an 8.1-fold increase (*p*-value < 0.01) in the mRNA-primed cohort and the subunit-primed cohort, respectively.

Up to one month post booster, the parental NAb titers elicited by nonadjuvanted CoV2 preS dTM (B.1.351) booster were all higher than the mean NAb titers of human convalescent sera (2.2 log_10_), and all AS03-adjuvanted candidate vaccines induced titers above the highest titer observed in human convalescent sera in the subunit-primed cohort (the lowest NAb titers were 3.6 log_10_ after the AS03-adjuvanted boost, and the highest NAb titer in human convalescent sera panel was 3.3 log_10_).

### Boosting with homologous and heterologous formulations of CoV2 preS dTM led to significant increase in neutralizing antibodies against the Beta variant, with a significant AS03-adjuvant effect

We next assessed NAb titers against the Beta variant using the lentivirus-based neutralization assay. After the primary immunization, Beta NAb titers were low to undetectable in the mRNA-primed cohort (mean titer of 1.4 log_10_) and low in the subunit-primed cohort (mean titer of 1.6 log_10_) at D35 (Fig. 1c). Seven months post-prime, Beta NAb titers were undetectable in the mRNA-primed cohort (<limit of quantification) and low to undetectable in the subunit-primed cohort (mean titer of 1.3 log_10_).

A booster dose with the non-adjuvanted CoV2 preS dTM (B.1.351) vaccine induced Beta NAb titers within 7 days after injection, with a mean titer of 2.6 log_10_ in the mRNA-primed and 2.9 log_10_ in the subunit-primed cohorts 14 days post-injection that were stable until D28, and then slightly decreased up to 3 months post-booster (mean titers of 2.3 log_10_ in both cohorts at D84).

The increase in Beta NAb titers from baseline was higher for the AS03-adjuvanted CoV2 preS dTM (B.1.351) vaccine candidate compared with non-adjuvanted CoV2 preS dTM (B.1.351) in the mRNA-primed cohort (8.5-fold, *p*-value = 0.057) and significantly higher in the subunit-primed cohort (11.8-fold, *p*-value < 0.001). No statistical differences were observed between the various AS03-adjuvanted vaccine formulations with mean Beta NAb titers of 3.3 log_10_ in the mRNA-primed animals (mean fold-increase of 180 from baseline) and 3.7 log_10_ in the subunit-primed animals (mean fold-increase of 280 from baseline) 14 days post-booster. In the two cohorts, after the AS03-adjuvanted booster immunization, the mean Beta NAb titers observed up to D28 were significantly higher than the peak titers post-primary vaccination on D35 whatever the vaccine formulations (*p*-values < 0.001, mean fold-increase of 46 in the mRNA-primed and 140 in the subunit-primed cohort). Over the 3-month period post-booster, the Beta NAb responses declined slightly in the AS03-adjuvanted groups, with mean titers of 2.5 log_10_ and 3.0 log_10_ in the mRNA- and subunit-primed cohorts, respectively, on D84.

### Boosting with CoV2 preS dTM led to significant increase in cross-neutralizing antibodies against Alpha, Gamma, Delta, Omicron and SARS-CoV-1, with a significant AS03-adjuvant effect

**Fig. 2:**
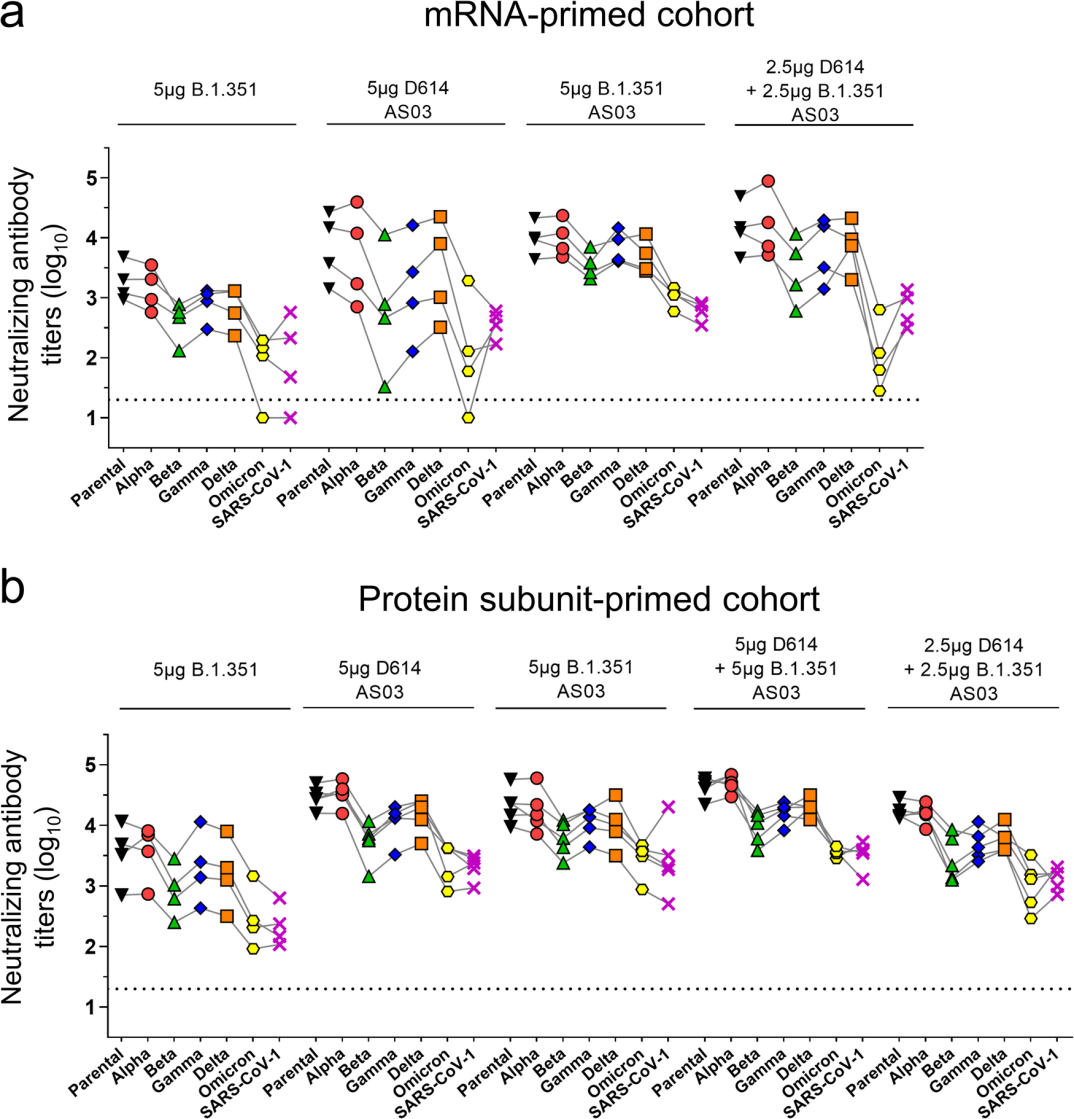
Booster cross-neutralizing antibody responses against the current VOC and SARS-CoV-1 in the mRNA- and subunit-primed macaques. Pseudovirus-neutralizing antibody assays against the parental SARS-CoV-2, variants of concern (Alpha, Beta, Gamma, Delta, and Omicron), and SARS-CoV-1 was assessed at day 14 after the booster injection in primed-macaques **a** mRNA-primed cohort (*n* = 4/group) and **b** protein subunit-primed cohort (*n* = 4 or 5/group). Individual macaque data are shown and are linked by connecting lines. Horizontal dotted lines indicate the limits of quantification of the assay.

The breadth of neutralization was further assessed against the other known SARS-CoV-2 VOC, Alpha, Gamma, Delta, Omicron, and SARS-CoV-1. NAb cross-reactivity against the VOC was observed 14 days post-injection with all vaccine formulations in both cohorts (Fig. 2, Supplementary Figs. 2 and  3).

The nonadjuvanted CoV2 preS dTM (B.1.351) vaccine booster induced, in the mRNA-primed cohort, mean NAb titers of 3.1 log_10_ (Alpha), 2.9 log_10_ (Gamma), 2.8 log_10_ (Delta), and 1.9 log_10_ (Omicron) (Fig. 2a) and, in subunit-primed cohort, mean VOC NAb titers of 3.5 log_10_ (Alpha), 3.0 log_10_ (Gamma) and 3.2 log_10_ (Delta), and 2.5 log_10_ (Omicron) (Fig. 2b).

Overall, the various AS03-adjuvanted vaccine boosters induced mean VOC NAb titers of 4.0 log_10_ (Alpha), 3.6 log_10_ (Gamma), 3.7 log_10_ (Delta), and 2.4 log_10_ (Omicron) (Fig. 2a) in the mRNA-primed cohort and mean VOC NAb titers of 4.4 log_10_ (Alpha), 4.0 log_10_ (Gamma) and 4.1 log_10_ (Delta), and 3.4 log_10_ (Omicron) (Fig. 2b) in the subunit-primed cohort. As observed with the parental strain and the Beta variant, a significant adjuvant effect was observed with the CoV2 preS dTM B.1.351 monovalent vaccine booster in both cohorts (*p*-value < 0.05) for Alpha, Gamma, Delta, and Omicron NAb titers (mean fold-increases in the mRNA-primed cohort: 7.4, 11.9, 7.1, and 13.8 respectively, and in the subunit-primed cohort: 5.7, 8.6, 6.6, and 12.6, respectively).

Unexpectedly, we also observed a significant enhanced neutralizing breadth against SARS-CoV-1 14 days post-injection with all booster-vaccine formulations (*p*-value < 0.001). The nonadjuvanted vaccine booster induced significant SARS-CoV-1 neutralizing titers in all macaques except one (mean NAb titers of 1.9 log_10_ and 2.3 log_10_ in the mRNA-primed and subunit-primed cohorts, respectively, *p*-value < 0.001). Overall, AS03-adjuvanted vaccine formulations induced significant SARS-CoV-1 NAb in all macaques, with mean titers of 2.7 log_10_ and 3.3 log_10_ in the mRNA-primed cohort and subunit-primed cohort, respectively (*p*-value < 0.001; Fig. 2 and Supplementary Fig. 4). The AS03 adjuvant effect was confirmed on SARS-CoV-1 NAb-titer increase from baseline when comparing the AS03-adjuvanted and the non-adjuvanted B.1.351 monovalent vaccine candidates (9.1-fold and 7-fold higher booster effect for mRNA-primed and subunit-primed cohort, respectively, *p*-value < 0.01).

### Booster immunization with CoV2 preS dTM led to higher functional to binding antibody ratio in both mRNA- and subunit-primed macaques

**Fig. 3:**
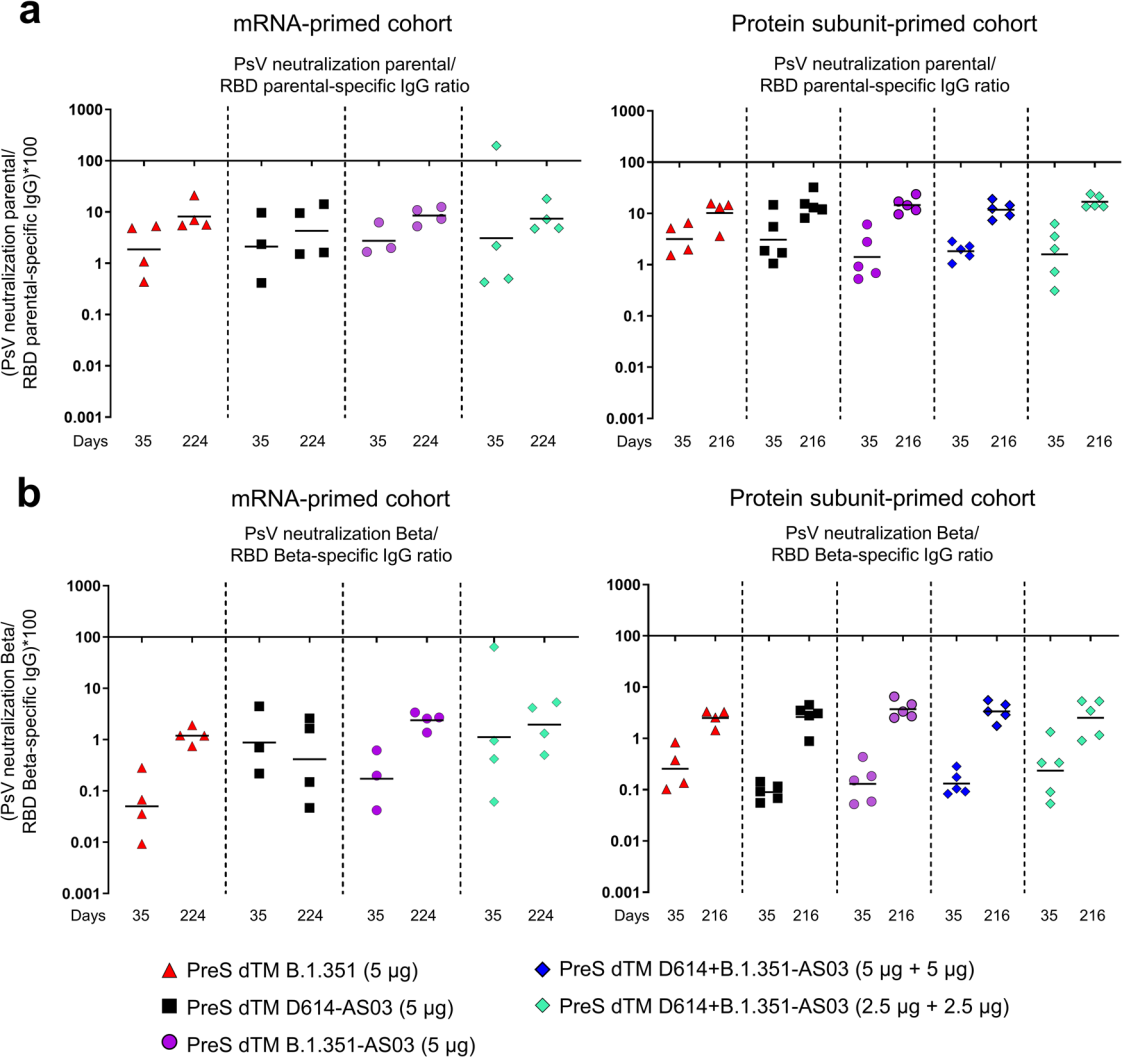
Booster immunization elicits an increase in functional antibody response in mRNA- and subunit-primed macaques. Pseudovirus NAb/RBD-binding IgG ratio for parental D614G (**a**) and for Beta (**b**), post prime (D35) and 2 weeks post-boost (D224 or D216) for mRNA-primed cohort (*n* = 4/group) and subunit-primed cohort (*n* = 4–5/group). Individual animals are shown; bars indicate means.

To better assess the impact of booster on the quality of antibody responses, we measured spike- and receptor-binding domain (RBD)-binding antibodies in sera collected at D35 post-prime and 14 days post-booster injection (Supplementary Fig. 5). We then calculated the individual ratio between neutralizing and RBD-binding antibodies for the parental strain and Beta variant (Fig. 3). The ratios increased in all groups, except for the Beta ratio in one group (CoV2 PreS dTM D614–AS03 monovalent group in the mRNA-primed cohort). The mean fold-increases for parental and Beta ratios reached 4.4- and 23-fold in the mRNA-primed cohort and reached 10- and 29-fold in the subunit-primed cohort, respectively. The consistent increase indicates that the booster dose increases functional antibody responses in both mRNA- and subunit-primed groups through induction of a higher proportion of neutralizing antibodies among RBD-specific antibodies.

## Discussion

We previously reported that our protein-based CoV2 preS dTM-AS03 subunit vaccine provided robust protection against challenge with SARS-CoV-2 in rhesus macaques and hamsters and provided robust immunity in humans. In this study, we show that a booster with various formulations of CoV2 preS dTM in macaques previously vaccinated with Sanofi’s mRNA (D614) or CoV2 preS dTM-AS03 (D614) subunit-vaccine candidates induced a robust increase in neutralizing antibody titers against the parental strain, as early as 7 days post-booster, and achieved significantly higher NAb titers than the D35 vaccination peak with AS03 adjuvant. Importantly, booster injection with CoV2 preS dTM formulations, induced cross-neutralization covering the five SARS-CoV-2 VOC, Alpha, Beta, Gamma, Delta, and Omicron, and broadened the NAb response to SARS-CoV-1. The VOC display from 10 (Alpha) to more than 30 (Omicron) mutations in the spike protein (Table 1), with some key mutations occurring in the RBD for VOC displaying the highest resistance to prior immunity (Beta, Gamma, and Omicron). In contrast, the SARS-CoV-1 spike protein displays about 350 amino acid differences compared with SARS-CoV-2 but still, was neutralized at similar levels as Omicron after the booster. The increase in neutralizing antibodies lasted over the 3-month follow-up period post-booster and was more pronounced on the neutralizing antibodies than on RBD-binding antibodies.

**Table 1:** Description of the reporter virus particles (RVPs) used in the pseudovirus neutralization assay.

Pango lineage	WHO name	Strain (Nextstrain)	Sequence source	Catalog number	Lot	Mutations relative to Wuhan D614
A	N/A	Wuhan (reference sequence) – D614	GenBank QHD43416.1	RVP-701	CG-113A	–
B.1	N/A	D614G	–	RVP-702G	CG-129A	D614G
B.1.1.7	alpha	20I/S:501Y.V1	QQH18545.1	RVP-706G	CG-135A	ΔH69/V70, ΔY144, N501Y, A570D, D614G, P681H, T716I, S982A, D1118H
B.1.351	beta	20H/S:501Y.V2	Tegally et al., 2020	RVP-724G	CG-180A	L18F, D80A, D215G, ΔL242/A243/L244, R246I, K417N, E484K, N501Y, D614G, A701V
P.1 or B.1.128	gamma	20 J/S:501Y.V3	QQX12069.1	RVP-708G	CG-160A	L18F, T20N, P26S, D138Y, R190S, K417T, E484K, N501Y, D614G, H655Y, T1027I, V1176
B.1.617.2	delta	21 A/S:478 K	cov-lineages.org	Custom	CG-233A	T19R, G142D, E156G, ΔF157/R158, L452R, T478K, D614G, P681R, D950N
B.1.529	omicron	21 K	EPI_ISL_6841980	RVP-768G	CG-296A	A67V, Δ69-70, T95I, G142D/Δ143-145, Δ211/L212I, ins214EPE, G339D, S371L, S373P, S375F, K417N, N440K, G446S, S477N, T478K, E484A, Q493R, G496S, Q498R, N501Y, Y505H, T547K, D614G, H655Y, N679K, P681H, N764K, D796Y, N856K, Q954H, N969K, L981F
NA	NA	SARS-CoV-1	P59594.1	RVP-801G	SG-115B	28% differences

*N/A* not applicable.

To our knowledge, this is the first report of SARS-CoV-2 booster-subunit vaccine formulations demonstrating broad cross-neutralizing antibodies covering the five VOC (Alpha, Beta, Gamma, Delta, and Omicron) in macaques primed with different vaccine platforms.

Furthermore, in our studies, we observed an extended breadth of neutralization to SARS-CoV-1, which was responsible for the more severe respiratory disease during the 2002–2003 outbreak^22^. Notably, the neutralizing titers induced against SARS-CoV-1 were similar to the titers against Omicron, although the SARS-CoV-1 spike bares only 72% homology with the SARS-CoV-2 spike. This confirms that the cross-neutralization is not a linear function of the number of mutations and suggests that Omicron might display the greatest antigenic distance from the parental D614 within SARS-CoV-2.

Although there was a trend for a more balanced neutralization profile with the monovalent B.1.351 or bivalent vaccine formulations, it is noteworthy that all booster-vaccine formulations tested induced NAb titers with similar breadth, suggesting that the booster vaccine mainly targets cross-reactive B cells as it was shown for mRNA-1273 booster vaccine in NHPs^23^.

We observed a significant adjuvant effect of AS03, which increased the titers of neutralizing antibodies against the parental strain, all VOC and SARS-CoV-1 when compared with nonadjuvanted formulation (6- to 8-fold on parental NAb titers, and 8- to 11.8-fold on Beta NAb titers). This is in line with previous findings in the context of pandemic influenza vaccination, where the AS03 adjuvant effect consistently resulted in increased magnitude and breadth of the response (including heterotypic antibody responses) in primed individuals^24–26^.

Interestingly, the non-adjuvanted B.1.351 monovalent formulation was also capable of boosting and broadening the neutralizing responses, suggesting that the AS03 adjuvant might not be as necessary for a booster compared with primary vaccination, where it was required to induce functional antibody responses. For comparison, the NAb titers induced by the non-adjuvanted monovalent B.1.351 vaccine were in the same range as those induced in NHPs with a third dose of mRNA-1273 vaccine for Beta, Gamma, and Delta variants measured using similar neutralization assays^27^.

The greater impact of the booster on the neutralization of VOC and SARS-CoV-1 compared with the neutralization of the parental strain suggests that the booster vaccine can remodel the antibody specificity in addition to increasing the antibody levels. Improvement of the antibody quality was also observed when calculating the ratios between functional (neutralizing) and RBD-binding antibodies (parental and Beta), which increased after the booster immunization. Broader neutralization, coupled to the higher proportion of neutralizing antibody after the booster, is probably due to activation of preexisting memory B cells in primed animals and further affinity maturation, possibly identifying a key role for memory B cells in mounting recall responses to SARS-CoV-2 antigens and generating cross-reactive NAbs^28^.

The stability of the NAb titers observed over 3 months after the booster immunization holds promise for an improved durability of the circulating antibodies.

As our macaques were not challenged, our study does not define mechanistic correlates of protection against SARS-CoV-2 variants, but various studies previously reported that NAb levels are highly predictive of immune protection from symptomatic SARS-CoV-2 infection^29–31^. Moreover, a recent report with mRNA vaccine established the critical role of NAbs as a correlate of protection against infection in NHPs^32^. Based on these studies, and on the levels of NAb titers observed here after the booster against the VOC and SARS-CoV-1, one can hypothesize that a booster with CoV2 preS dTM vaccine will augment and extend the protection against existing and newly emerging SARS-CoV-2 variants. In particular, AS03-adjuvanted CoV2 preS dTM booster immunization resulted in higher neutralizing titers than those observed in NHPs with a third dose of mRNA-1273 vaccine for the Beta, Gamma, and Delta variants^27^, hinting to a beneficial effect of an adjuvanted subunit booster strategy for protection, especially against distant variants such as Omicron, and, potentially, durability of the response.

These data have important implications for the utility of current vaccines and inform boosting strategies to address the risks associated with SARS-CoV-2 VOC and waning immunity. Based on these encouraging results, non-adjuvanted and AS03-adjuvanted vaccine formulations, using a dose range of antigen and adjuvant, are being evaluated as booster vaccines in a clinical trial, and the preliminary results confirm our observations in primed NHPs^33^.

The study has several limitations. First, the study included a small number of animals, specifically 4–5 macaques per group. Second, as adenovirus-vector vaccine-primed macaques were not available, the booster was evaluated in mRNA- and subunit-primed macaques only. However, the adenovirus priming platform is included in the clinical trial (NCT04762680). Third, the interval between the primary vaccination and the booster was set at 7 months, evaluating shorter and longer intervals from 4 to 10 months is part of the clinical trial. Finally, the mechanism underlying the cross-neutralization conferred by the booster, i.e., broadening of the repertoire of antibodies or selection of preexisting cross-reactive specificities, would need to be further defined. While the antibody repertoire is not easily accessible due to limitation of the immunological tools in macaques, analysis of the Ab quality using system serology, epitope mapping, and memory B-cell population at later timepoints after the booster immunization is ongoing to better characterize the mechanism for cross-protection.

In conclusion, a single dose of the CoV2 preS dTM recombinant vaccine (non-adjuvanted monovalent B.1.351, AS03-adjuvanted monovalent D614 or B.1.351, or bivalent D614 + B.1.351) used as a booster in macaques previously vaccinated with mRNA or subunit COVID-19 vaccines induced high and persistent cross-reactive neutralizing antibodies against the currently known SARS-CoV-2 variants of concern (Alpha, Beta, Gamma, Delta, and Omicron) and the two-decade-old SARS-CoV-1, as early as 7 days post-boost injection. Our findings show that CoV2 preS dTM vaccine candidates when used as a booster have the potential to offer cross-protection against a broad spectrum of variants and have important implications for vaccine control of SARS-CoV-2 VOC. Preliminary results in humans with monovalent CoV2 preS dTM-AS03 (D614) vaccine booster in subjects previously primed with adenovirus-vector-based and mRNA-based COVID-19 vaccines^33^ confirm the value of the primed-NHP model used here.

## Methods

### Vaccines

For the primary immunization, the mRNA vaccines were SARS-CoV-2 prefusion spike constructs 2 P, GSAS, 2 P/GSAS, 2 P/GSAS/ALAYT and 6 P/GSAS described in Kalnin et al.^34^, the subunit vaccines were AS03-adjuvanted CoV2 preS dTM vaccines, where the antigens were produced using the phase-I/-II manufacturing process, 1.3- and 2.6-µg doses, or using an intermediate manufacturing process, 2.4 µg dose.

For the booster, the CoV2 preS dTM derived from the parental D614 strain and the B.1.351 variant was produced using an optimized purification process to ensure a minimum of 90% purity.

The antigens were formulated in monovalent or bivalent formulations with AS03 adjuvant, the monovalent CoV2 preS dTM (B.1.351) was also formulated without AS03. The CoV2 preS dTM was produced from a Sanofi proprietary cell culture technology based on the insect cell—baculovirus system, referred to as the Baculovirus Expression Vector System (BEVS). The CoV2 preS dTM (D614) sequence was designed based on the Wuhan YP_009724390.1 strain S sequence, modified with 2 prolines in the S2 region, deletion of the transmembrane region, and addition of the T4 foldon trimerization domain. The CoV2 preS dTM (B.1.351) was designed based on the B.1.351 sequence (GISAID Accession EPI_ISL_1048524) and contains the same modifications.

AS03 is a proprietary adjuvant system composed of α-tocopherol, squalene, and polysorbate 80 in an oil-in-water emulsion manufactured by GSK. Vaccine doses were formulated by diluting the appropriate dose of preS dTM with PBS-tween to 250 µL, then mixing with 250 µL of AS03, followed by inversion five times for a final volume of 500 µL. Each dose of AS03 contains 11.86 mg of α-tocopherol, 10.69 mg of squalene, and 4.86 mg of polysorbate-80 (Tween 80) in PBS.

### Animals and study design

Animal experiments were carried out in compliance with all pertinent US National Institutes of Health regulations and were conducted with approved animal protocols from the Institutional Animal Care and Use Committee (IACUC) at the research facilities. NHP studies were conducted at the University of Louisiana at Lafayette New Iberia Research Center.

Two cohorts of vaccinated NHPs received a booster immunization after randomizing each group within a cohort based on their baseline characteristics (Fig. 1a).

In the mRNA-primed cohort, 16 adult male and female Mauritius cynomolgus macaques (*Macaca fascicularis*) aged 4–10 years, selected based on their responses to the primary vaccination, were randomly allocated to 4 groups of 4 animals according to their baseline characteristics.

In the subunit-primed cohort, 24 adult-male Indian rhesus macaques (*Macaca mulatta*) aged 4–7 years were randomly allocated to 5 groups of 4 or 5 animals. In the priming phase, animals received two immunizations of either Sanofi’s mRNA COVID (D614) experimental candidate vaccines or CoV2 preS dTM-AS03 (D614) vaccine through the intramuscular route in the deltoid at day 0 and day 21. Seven months after the primary immunization, both cohorts were immunized with CoV2 preS dTM (B.1.351) without adjuvant, CoV2 preS dTM (D614)–AS03, CoV2 preS dTM (B.1.351)–AS03, and a bivalent CoV2 preS dTM (D614 + B.1.351)–AS03. All groups received a total dose of 5 µg of CoV2 preS dTM antigen. An additional group in subunit-primed cohort received a bivalent CoV2 preS dTM (D614 + B.1.351)–AS03 high-antigen dose (5 + 5 µg). All immunologic analyses were performed blinded on serum collected at 7, 14, 21, 28, 56, and 84 days post-boost injection. Animal studies were conducted in compliance with all relevant local, state, and federal regulations, and were approved by the New Iberia Research Center.

### Convalescent human sera

Convalescent serum panel (*N* = 93) was obtained from commercial vendors (Sanguine Biobank, iSpecimen, and PPD). The serum samples were collected within 3 months following PCR-positive diagnosis of COVID-19.

The WHO International Standard for anti-SARS-CoV-2 immunoglobulin (human) (NIBSC code: 20/136) was also used.

### Pseudovirus-based virus neutralization assays

Serum samples were diluted 1:4 or 1:20 in media (FluoroBrite™ phenol-red-free DMEM + 10% FBS + 10 mM HEPES + 1% PS + 1% GlutaMAX™) and heat-inactivated at 56 °C for 30 min. Further, 2-fold, 11-point, dilution series of the heat-inactivated serum were performed in media. Diluted serum samples were mixed with reporter-virus particle (RVP)-GFP (Integral Molecular) listed in Table 1 diluted to contain ~300 infectious particles per well and incubated for 1 h at 37 °C. 96-well plates of ~50% confluent 293T-hsACE2 clonal cells (Integral Molecular, Cat# C-HA102) in 75 µL volume were inoculated with 50 µL of the serum + virus mixtures and incubated at 37 °C for 72 h. At the end of the 72-hour incubation, plates were scanned on a high-content imager and individual GFP-expressing cells were counted. The neutralizing antibody titer was reported as the reciprocal of the dilution that reduced the number of virus plaques in the test by 50%.

### Enzyme linked immunosorbent assay (ELISA)

Nunc microwell plates were coated with SARS-CoV S-GCN4 protein (GeneArt, expressed in Expi 293 cell line) at 0.5 µg/mL or SARS-CoV2 variant RBD protein purchased from SinoBiological (D614: 40592-v08h; Beta: 40592-v08h85; Delta: 40592-v08h90; SARS-CoV-1: 10583CV100) at 1 µg/mL in PBS at 4 °C for overnight. Plates were washed 3 times with PBS–Tween 0.1% before blocking with 1% BSA in PBS-Tween 0.1% for 1 h. Samples were heat-inactivated at 56 °C for 30 min and plated at a 1:450 initial dilution followed by 3-fold, 7-point serial dilutions in blocking buffer. Plates were washed 3 times after a 1-hour incubation at room temperature before adding 50 µL of 1:8000 Goat anti-human IgG (Jackson Immuno Research, CAT# 109-036-098) to each well. Plates were incubated at room temperature for 1 h and washed thrice. Plates were developed using Pierce 1-Step Ultra TMB-ELISA Substrate Solution for 6 min and stopped by TMB STOP solution. Plates were read at 450 nm in SpectraMax^®^ plate reader, and the data analyzed using Softmax^®^ Pro 6.5.1 GxP software and the proprietary software, Sanofi Universal Exporter 2.1. Antibody titers were reported as the highest dilution that is equal to 0.2-OD cutoff.

### Statistical analyses

For both, mRNA-primed and CoV2 preS dTM-AS03-primed cohorts, at the time of the assignment, the characteristics at baseline (sex, age, and weight) were balanced to have comparable groups. The pseudovirus-neutralizing titers were also taken into account as well as the previous vaccine groups. ELISA titers and neutralizing titers were log_10_-transformed prior to statistical analysis. All statistical tests were two-sided, and the nominal level of statistical significance was set to *α* = 5%. The analyses were performed on SEG SAS v9.4^®^.

To use the ANOVA models, the normality of the residual of the model was checked using Normal Probability Plot, and the results were considered acceptable. Although the number of animals is small, we assumed that the readouts analyzed (IgG and pseudovirus-neutralizing titers) follow a log-normal distribution based on past experience. The biostatisticians concluded that the use of ANOVA test was thus acceptable. Since nonparametric tests allow to conclude only on the median or on the rank, one-way ANOVA test was preferred to estimate and compare the mean fold-changes from baseline between the different groups.

Statistical comparisons were performed among different groups and conducted as follows for each readout and each strain separately: one way ANOVA with the group as factor was performed to analyze the IgG ratio, PsV ratio post-booster, and post prime. Mixed-effect models for repeated measures, including group, day, and their interactions, where day was specified as repeated measure, were used for PsV neut/IgG ratio and to evaluate the boost effect.

Using the data pooled from both cohorts (i.e., primed with mRNA or protein subunit), a concordance analysis was performed between the D614G and D614 PsV-neutralizing titers using a regression. The concordance was considered verified if the 90% confidence interval of the slope is included in [0.80; 1.25].

### Reporting summary

Further information on research design is available in the Nature Research Reporting Summary linked to this article.

## Supplementary information

Supplementary Information

Reporting Summary

## Data Availability

Accession codes for the publicly available datasets are provided in the paper. The source data generated in this study are provided as Supplementary Material. Source data are provided with this paper.

## Source data

Source Data
