# Supplementary material for: Protein-based SARS-CoV-2 spike vaccine booster increases cross-neutralization against SARS-CoV-2 variants of concern in non-human primates
Source: Nat Commun. 2022 Mar 31;13:1699. doi: 10.1038/s41467-022-29219-2 (PMC8971430; doi:10.1038/s41467-022-29219-2)
Supplement: Supplementary file 1 — Supplementary Information [file 41467_2022_29219_MOESM1_ESM.pdf]

Pavot *et al.* Supplementary Figures

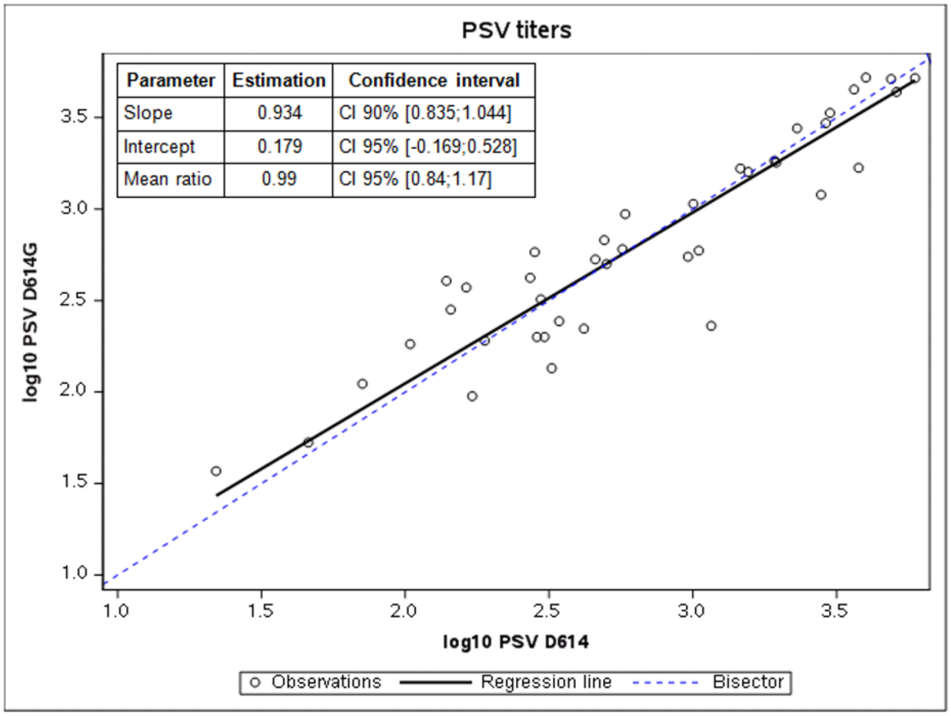

Supplementary Fig. 1. Concordance analysis of pseudovirus neutralization using D614 or D614G pseudovirus.

Forty samples were analyzed. The concordance is verified between D614 and D614G PsV neutralization: slope = 0.934 (CI 90% [0.835;1.044])

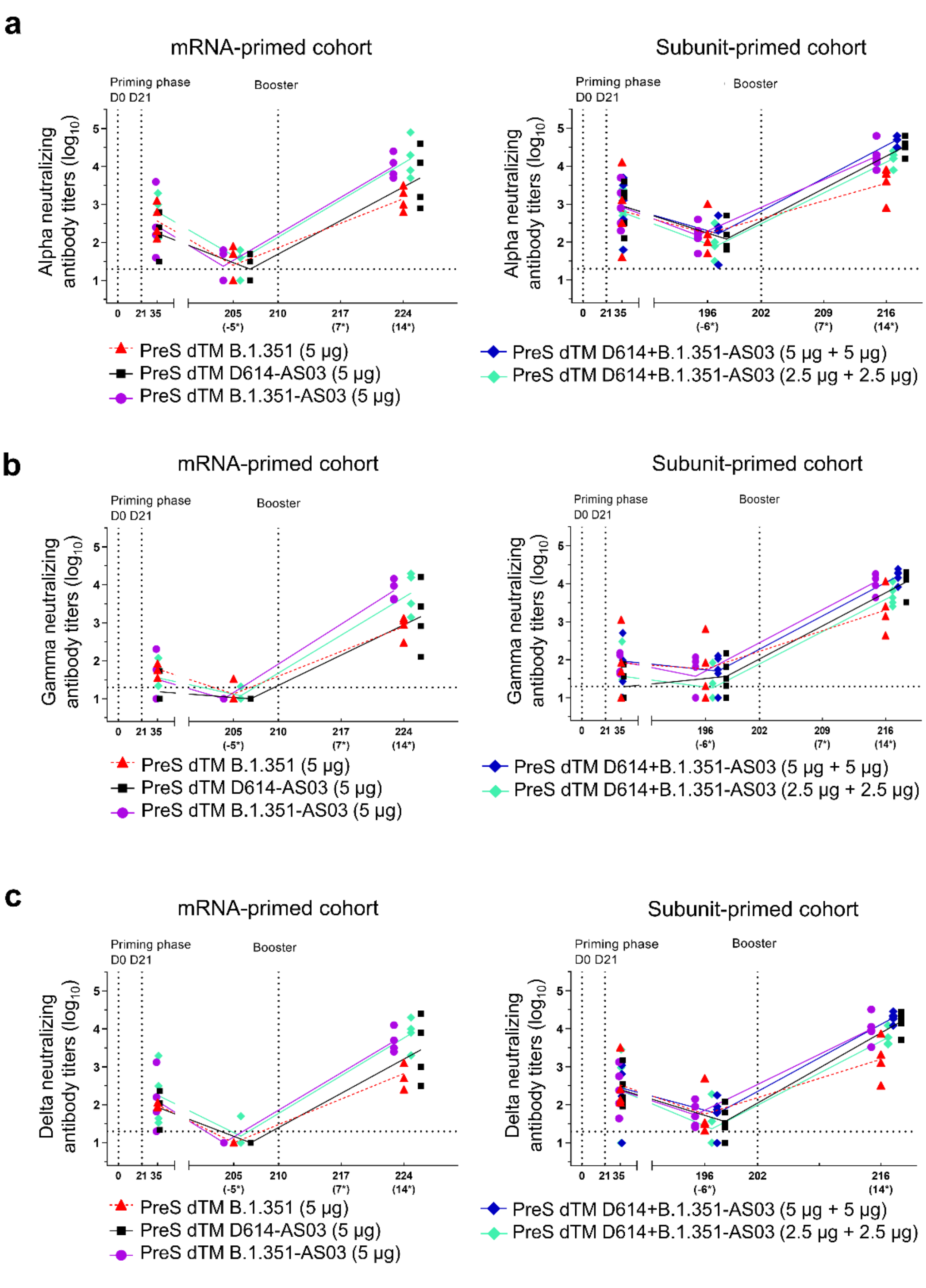

Supplementary Fig. 2. Booster cross-neutralizing antibody responses against Alpha, Gamma and Delta variants in the mRNA- and subunit-primed macaques. Pseudovirus neutralizing antibody (NAb) titers against **a** the SARS-CoV-2 Alpha , **b** Gamma and **c** Delta variants were assessed at D35 and 7 months after the priming phase and 2 weeks after the booster immunization in macaques. Individual macaque data are shown (n=4 to 5/group). Connecting lines

indicate mean responses and horizontal dotted lines the limits of quantification of the assay. Asterisks = timepoints relative to boosters.

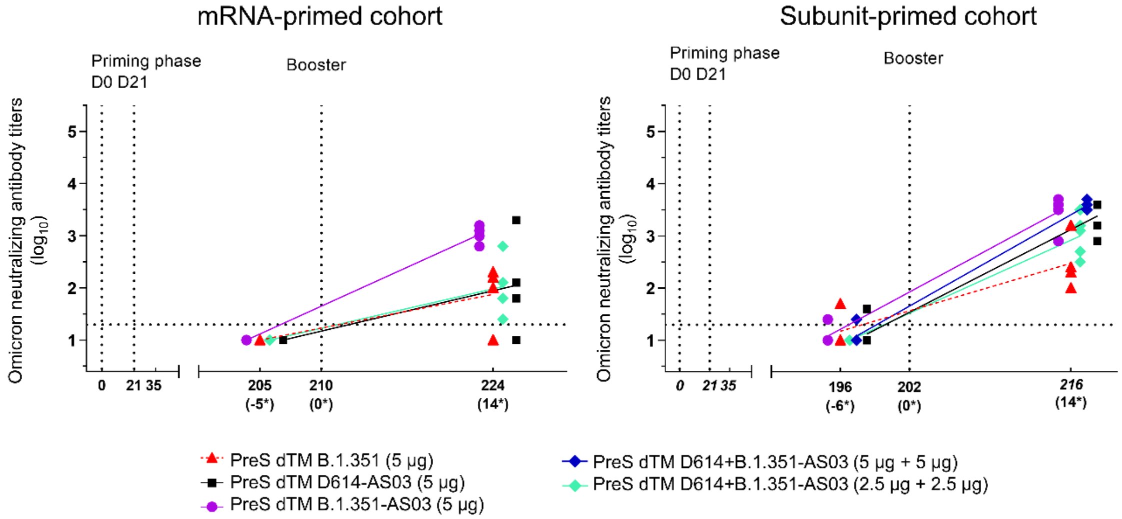

Supplementary Fig. 3. Booster cross-neutralizing antibody responses against Omicron variant in the mRNA- and subunit-primed macaques. Pseudovirus neutralizing antibody (NAb) titers against Omicron were assessed 7 months after the priming phase and 2 weeks after the booster immunization in macaques. Individual macaque data are shown (n=4 to 5/group). Connecting lines indicate mean responses and horizontal dotted lines the limits of quantification of the assay. Asterisks = timepoints relative to boosters.

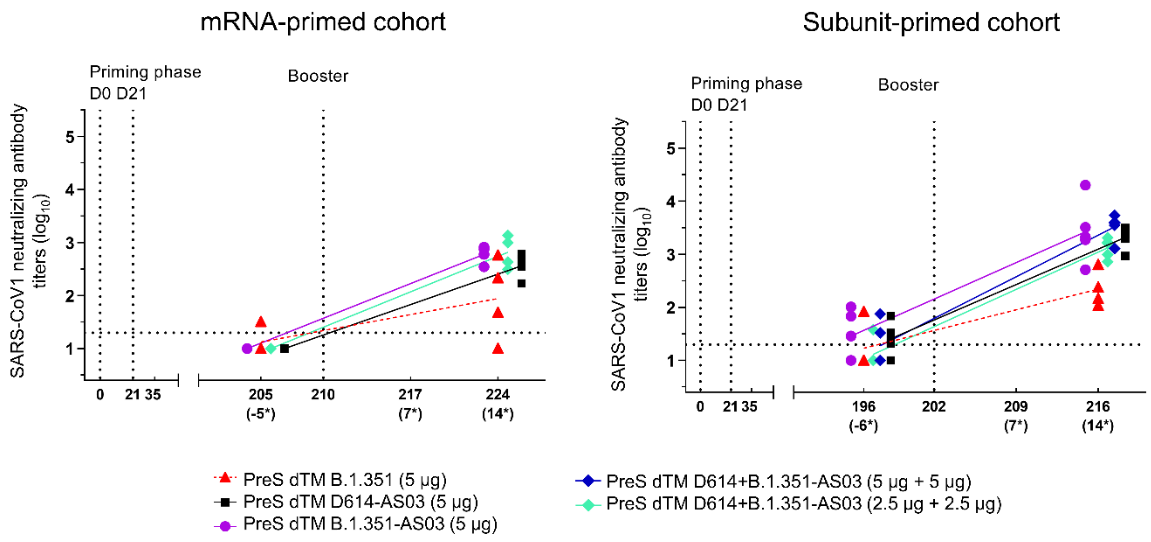

Supplementary Fig. 4. Booster cross-neutralizing antibody responses against SARS-CoV-1 in the mRNA- and subunit-primed macaques. Pseudovirus neutralizing antibody (NAb) titers against SARS-CoV-1 were assessed 7 months after the priming phase and 2 weeks after the booster immunization in macaques. Individual macaque data are shown (n=4 to 5/group). Connecting lines indicate mean responses and horizontal dotted lines the limits of quantification of the assay. Asterisks = timepoints relative to boosters.

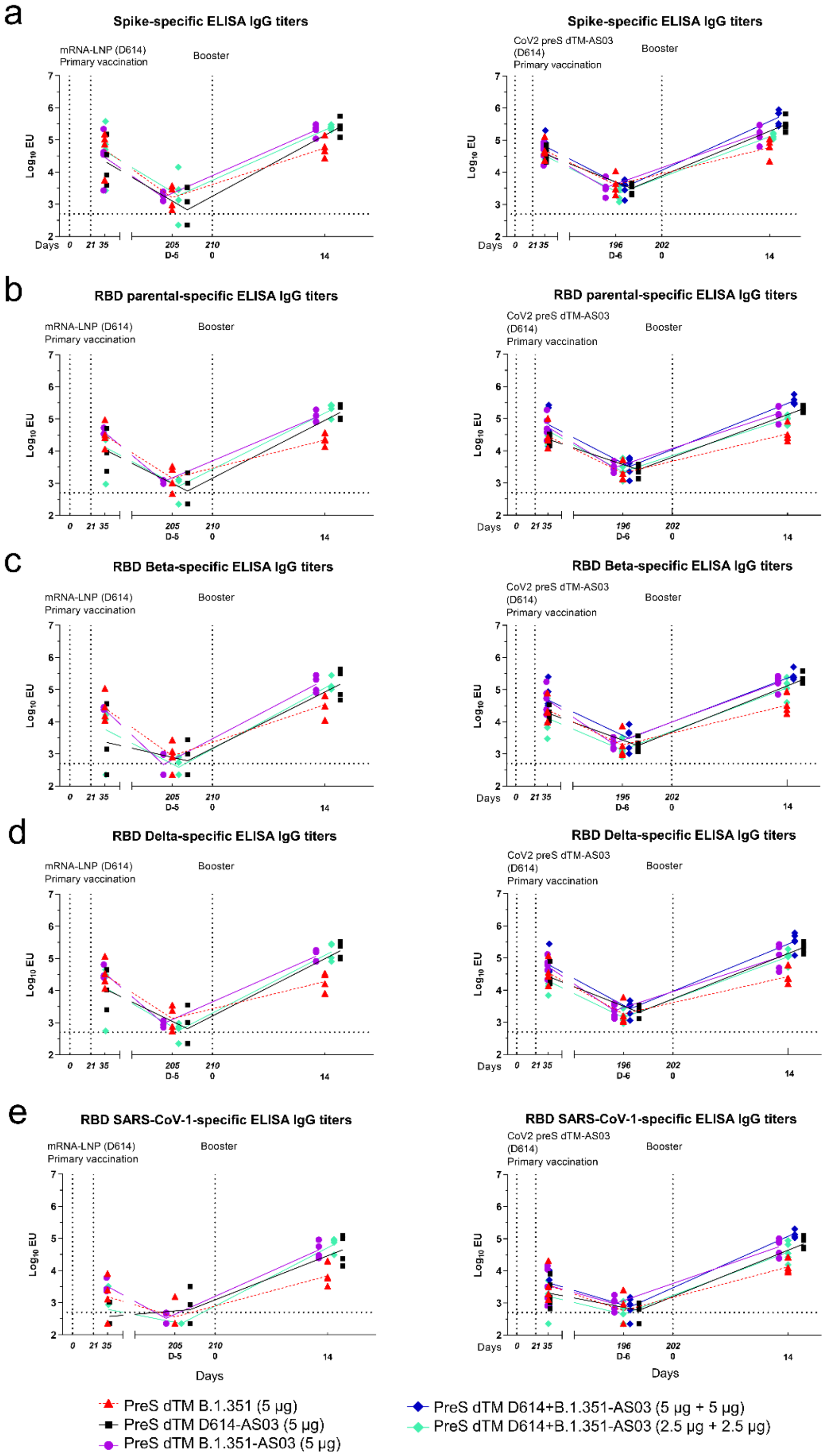
a
Spike-specific ELISA IgG titers
mRNA-LNP (D614)
Primary vaccination
Booster
CoV2 preS dTM-AS03
(D614)
Primary vaccination
b
RBD parental-specific ELISA IgG titers
c
RBD Beta-specific ELISA IgG titers
d
RBD Delta-specific ELISA IgG titers
e
RBD SARS-CoV-1-specific ELISA IgG titers
Log10 EU
Days
0
21
35
205
D-5
210
0
14
196
D-6
202
PreS dTM B.1.351 (5 µg)
PreS dTM D614-AS03 (5 µg)
PreS dTM B.1.351-AS03 (5 µg)
PreS dTM D614+B.1.351-AS03 (5 µg + 5 µg)
PreS dTM D614+B.1.351-AS03 (2.5 µg + 2.5 µg)

Supplementary Fig.5. Antibody responses in vaccinated rhesus macaques. a Full-length spike-specific binding antibody responses were assessed by ELISA. RBD-specific binding antibody responses against **b** Parental, **c** Beta, **d** Delta, and **e** SARS-CoV-1 were assessed by ELISA. Individual macaque data are shown (n=4 to 5/group). Connecting lines indicate mean responses and horizontal dotted lines the limits of quantification of the assay.
